# Efficacy and Safety of Duloxetine with Gabapentin or Amitriptyline Versus Duloxetine Monotherapy in Chemotherapy-Induced Peripheral Neuropathy: Randomized Controlled Trial

**DOI:** 10.3390/ph19040553

**Published:** 2026-03-30

**Authors:** Hager Salah, Ahmed Hassan Shaaban, Mona A. Abdelrahman, Hasnaa Osama, Asmaa M. El-Kalaawy

**Affiliations:** 1 Department of Clinical Pharmacy Services, King Hamad University Hospital, Royal Medical Services, Busaiteen P.O. Box 24343, Bahrain; 2 Department of Clinical Oncology, Faculty of Medicine, Beni-Suef University, Beni-Suef 62511, Egypt; ahmedhassan_dr@yahoo.com; 3 Department of Clinical Pharmacy, Faculty of Pharmacy, Beni-Suef University, Beni-Suef 62511, Egypt; dr_mona_2008@yahoo.com (M.A.A.); hasnaa_osama2010@yahoo.com (H.O.); 4 Department of Pharmacology, Faculty of Medicine, Beni-Suef University, Beni-Suef 62511, Egypt; asmaa.hussein@med.bsu.edu.eg

**Keywords:** amitriptyline, duloxetine, gabapentin, CIPN, chemotherapy

## Abstract

**Introduction**: Chemotherapy-induced peripheral neuropathy (CIPN) is a dose-limiting toxicity affecting many patients treated with neurotoxic agents, leading to persistent pain and impaired quality of life. **Methods**: In our trial, Trial ID: NCT06091553, 160 patients met the eligibility criteria and were randomized into three groups. First, Arm D (duloxetine). Second, Arm (D + A): duloxetine is augmented with amitriptyline. Third, Arm (D + G): duloxetine is augmented with gabapentin. The primary outcome is the difference in Pain Inventory—Short Form (BPI-SF) measured during the final follow-up week (Week 4 and Week 8) between the treatments. **Results**: All groups showed significant within-group reductions in pain scores from baseline to Weeks 4 and 8. Meanwhile, all groups exhibited numerical improvements for the average pain by Week 8. No statistically significant differences were found between groups at either Week 4 (*p* = 0.161) or Week 8 (*p* = 0.868). Similarly, the proportion of responders was comparable across treatment arms at both time points, with 74.5–82.8% achieving a clinically meaningful reduction in pain by Week 8 (*p* = 0.566). **Conclusions**: These findings support duloxetine as an evidence-based first-line therapy for painful CIPN, while combination regimens may be reserved for individualized use in patients with inadequate response, pending confirmation in larger multicenter trials.

## 1. Introduction

Chemotherapy-induced peripheral neuropathy, also known as CIPN, is primarily a sensory degeneration of nerves. This can additionally manifest as changes in autonomic and motor function [1].

The most prominent adverse effects caused by several chemotherapy drugs, including cisplatin, oxaliplatin, paclitaxel, vincristine, and bortezomib, are off target in nature. CIPN is the most prevalent among these negative consequences [2]. A significant loss of primary afferent sensory axonal fibers is one of the defining characteristics of CIPN, which ultimately results in sensory abnormalities in patients. It is estimated that up to 75 percent of patients acquired CIPN while they were undergoing chemotherapy [3]. The lack of neuroprotective measures and the limited treatment choices frequently necessitate a reduction in the amount of life-saving chemotherapy or even an early termination of the treatment, which has an impact on the therapeutic efficacy and the patient’s ability to survive [4]. CIPN can occur in a variety of ways, with the overall dosage and type of medication used having a major impact. In general, around 60% to 80% of patients are documented as having CIPN symptoms in the month following the end of neurotoxic chemotherapy [1]. CIPN is mainly characterized by sensory axonal peripheral neuropathy [5]. This condition often manifests in a pattern resembling a stocking and glove. The distribution of the condition primarily affects longer axons, with the longer axons being impacted [6]. Patients with this condition primarily experience abnormal sensory perception, which can lead to balance issues and unstable walking in addition to sensations including a lack of sensation, heat, tingling, or pain in the extremities [7]. While the symptoms may eventually go away, many individuals often have them for the rest of their lives [8]. It was believed until a few years ago that CIPN seldom resulted in substantial restrictions on individuals impacted [9].

Unfortunately, neuropathy has the potential to have negative impacts on the patient’s general well-being and standard of life [10]. CIPN has been associated with several adverse consequences, including fatigue, psychological distress, and a reduction in physical independence, according to research [11,12].

According to the recommendations for clinical practice that were recently issued by the American Society of Clinical Oncology (ASCO), there are no therapeutic options that are strongly recommended for the purpose of avoiding or dealing with chemotherapy-induced peripheral neuropathy (CIPN) that has already occurred except for duloxetine [13].

The only suggested method of reducing neurotoxicity is still dose reduction because there are few neuroprotective and therapeutic options [13]. As a result, clinical and survival outcomes may be impacted by the tolerance of an effective anti-cancer medication being decreased, impacting clinical and survival outcomes [14]. Given that neurotoxic therapies for malignancies sometimes include considering the rising rates of survival, many cancer survivors are probably going to experience long-term results of CIPN [15]. The studies demonstrated that gabapentin administration suppressed chemotherapy-induced mechanical hyperalgesia, whereas duloxetine reduced cold allodynia. The mechanism involved the inhibition of ERK1/2 phosphorylation in the spinal cord, which is a critical intracellular signaling pathway [16]. Also, a new study suggested that amitriptyline acts on NMDA receptors in two ways: it increases Ca-dependent desensitization and blocks trapping channels; this suggested an add-on mechanism as a role in CIPN [17].

However, it remains unclear whether adding other treatments to duloxetine provides superior analgesia. This study aimed to compare the efficacy and safety of duloxetine augmented with gabapentin, and amitriptyline augmented with duloxetine vs. duloxetine alone, in chemotherapy-induced neuropathy in cancer patients. To our knowledge this is the first trial to assess add-on therapy to duloxetine to reduce CIPN.

## 2. Results

### 2.1. Baseline Characteristics of the Study Sample

The baseline demographic, clinical, and treatment-related characteristics of 160 patients with moderate-to-severe chemotherapy-induced peripheral neuropathy (CIPN), stratified by the treatment group. The mean age was 48.9 ± 13.6 years, with a predominance of females (61.9%), and no statistically significant differences in age, gender, BMI, serum creatinine, hemoglobin, or leukocyte count across groups (all *p* > 0.05). Most patients had Grade 1 neuropathy (68.1%), while Grade 3 neuropathy was more frequent in the duloxetine monotherapy group (23.4%) compared to the combination groups. Co-existing diseases were significantly more prevalent in the duloxetine + amitriptyline group (31%) compared to the other groups (*p* = 0.024)—all represented in Table 1.

Breast and colo-rectal cancers were the most common types, and over three-quarters of patients had stable disease. Most patients received either platinum-based (47.5%) or Taxane-based (42.5%) chemotherapy. The duration of chemotherapy differed significantly between groups (*p* = 0.042), with a higher proportion of patients in the duloxetine + gabapentin group receiving treatment for less than six months. Radiation therapy was also more frequently used in the duloxetine monotherapy group (63.8%) compared to others (*p* = 0.004). Other variables, including cancer stage, metastasis, treatment initiation with chemotherapy, prior surgery, analgesic use, and B12 supplementation, were comparable across groups.

### 2.2. Changes in Pain Intensity, Pain Relief, and Response Rates over 8 Weeks by Treatment Group

**Figure 1:**
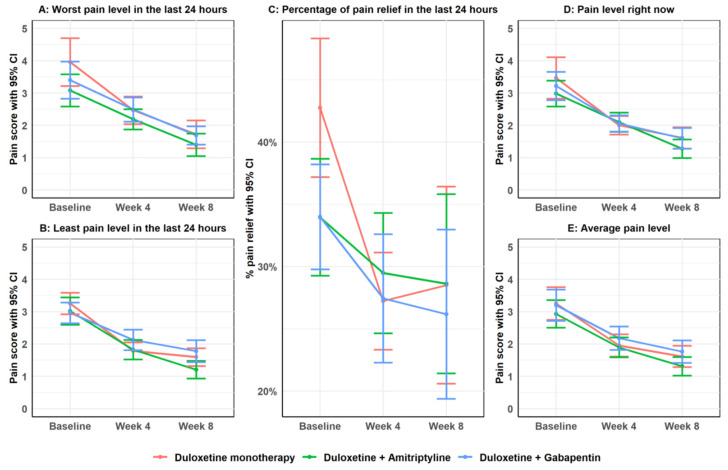
Changes in pain intensity and pain relief over 8 weeks in patients with moderate-to-severe chemotherapy-induced peripheral neuropathy receiving duloxetine alone or in combination with amitriptyline or gabapentin.

Figure 1 illustrates the changes in various dimensions of pain intensity and perceived pain relief from baseline to Week 8 among patients with moderate-to-severe chemotherapy-induced peripheral neuropathy (CIPN), stratified by treatment group. All three treatment arms (duloxetine monotherapy, duloxetine + amitriptyline, and duloxetine + gabapentin) demonstrated significant within-group improvements in worst pain, least pain, average pain, and current pain levels (panels A, B, D, and E, respectively), as confirmed by GEE analyses in Supplementary Table S1 (*p* < 0.001 for all comparisons to baseline). Duloxetine monotherapy was associated with the greatest absolute reductions in both average and current pain, whereas the addition of amitriptyline or gabapentin yielded comparable improvements by Week 8. In contrast, changes in reported pain relief over 24 h (panel C) were variable between groups, with a statistically significant improvement observed in the duloxetine monotherapy and duloxetine + gabapentin arms.

Between-group comparisons are presented in Supplementary Table S2, which reports both unadjusted and covariate-adjusted generalized estimating equation (GEE) models. After adjustment for the baseline variables that showed imbalance between groups in Table 1 (co-existing disease, chemotherapy duration >6 months, and receipt of radiotherapy), the results remained largely consistent with the unadjusted analyses. Across all pain dimensions and time points, no statistically significant between-group differences remained after Bonferroni correction. Unadjusted comparisons suggested a potential advantage for the duloxetine + amitriptyline group compared with duloxetine + gabapentin for least pain in the last 24 h at Week 8 (MD = −0.57, 95% CI −1.00 to −0.15, *p* = 0.008) and average pain at Week 8 (MD = −0.45, 95% CI −0.89 to −0.01, *p* = 0.043). However, these differences were attenuated after Bonferroni correction and were no longer statistically significant. The adjusted GEE models showed similar patterns, with effect estimates and confidence intervals closely aligned with the unadjusted results, indicating that the baseline imbalances did not materially influence the treatment comparisons. Overall, these analyses support that all three treatment regimens produced clinically meaningful improvements in pain intensity over time, with no regimen demonstrating a clearly superior between-group efficacy after accounting for multiple testing and baseline covariate imbalances.

In addition to the continuous pain outcomes, Supplementary Table S3 summarizes the percentage reduction in average pain scores and the proportion of responders (defined as ≥30% reduction from baseline) at Weeks 4 and 8 across the three treatment groups. While all groups exhibited numerical improvements in average pain by Week 8, the mean percentage reduction varied considerably, with high standard deviations reflecting inter-individual variability. No statistically significant differences were found between groups at either Week 4 (*p* = 0.161) or Week 8 (*p* = 0.868). Similarly, the proportion of responders was comparable across treatment arms at both time points, with 74.5–82.8% achieving a clinically meaningful reduction in pain by Week 8 (*p* = 0.566). These findings reinforce the comparable effectiveness of the three treatment regimens in achieving clinically relevant pain relief over time.

Mean scores (with 95% confidence intervals) for five pain-related outcomes are plotted over time for each treatment group: duloxetine monotherapy (red), duloxetine + amitriptyline (D + A) (green), and duloxetine + gabapentin (D + G) (blue). Panels A–E represent worst pain, least pain, average pain, current pain, and percentage of pain relief, respectively, measured using the Brief Pain Inventory—Short Form (BPI-SF). All groups showed significant within-group reductions in pain scores from baseline to Weeks 4 and 8 (see Supplementary Table S1). Duloxetine monotherapy was associated with the greatest absolute reductions in both average and current pain, whereas the addition of amitriptyline or gabapentin yielded comparable improvements by Week 8. However, no statistically significant differences were observed between treatment groups (D + A) or (D + G) at any time point after Bonferroni adjustment (see Supplementary Table S2).

### 2.3. Predictors of Insufficient Pain Response at Week 8

Table 2 summarizes the results of simple and multiple logistic regression analyses for predictors of insufficient analgesic response at the end of the 8-week follow-up. In the adjusted multiple regression model, patients receiving duloxetine + amitriptyline had significantly lower odds of insufficient response compared to duloxetine monotherapy (aOR = 0.23, 95% CI: 0.05–0.89, *p* = 0.039). Factors associated with reduced odds of insufficient response included having a family history of neuropathy (aOR = 0.13, 95% CI: 0.02–0.58, *p* = 0.014), regression of cancer status (aOR = 0.01, 95% CI: 0.00–0.24, *p* = 0.009), starting treatment with chemotherapy (aOR = 0.05, 95% CI: 0.01–0.23, *p* < 0.001), and chemotherapy duration >6 months (aOR = 0.09, 95% CI: 0.01–0.47, *p* = 0.008).

Conversely, higher odds of insufficient response were observed in patients with stage III disease (aOR = 10.03, 95% CI: 1.51–83.33, *p* = 0.022), stage IV disease (aOR = 52.75, 95% CI: 1.49–2266.13, *p* = 0.030), Taxane-based chemotherapy (aOR = 9.05, 95% CI: 1.38–79.01, *p* = 0.032), other chemotherapy classes (aOR = 24.61, 95% CI: 1.92–428.76, *p* = 0.019), and prior surgery (aOR = 7.92, 95% CI: 1.20–65.40, *p* = 0.041). Other demographic and clinical variables were not significantly associated with treatment response after adjustment.

### 2.4. Time to Clinically Meaningful Pain Reduction by Treatment Group

**Figure 2:**
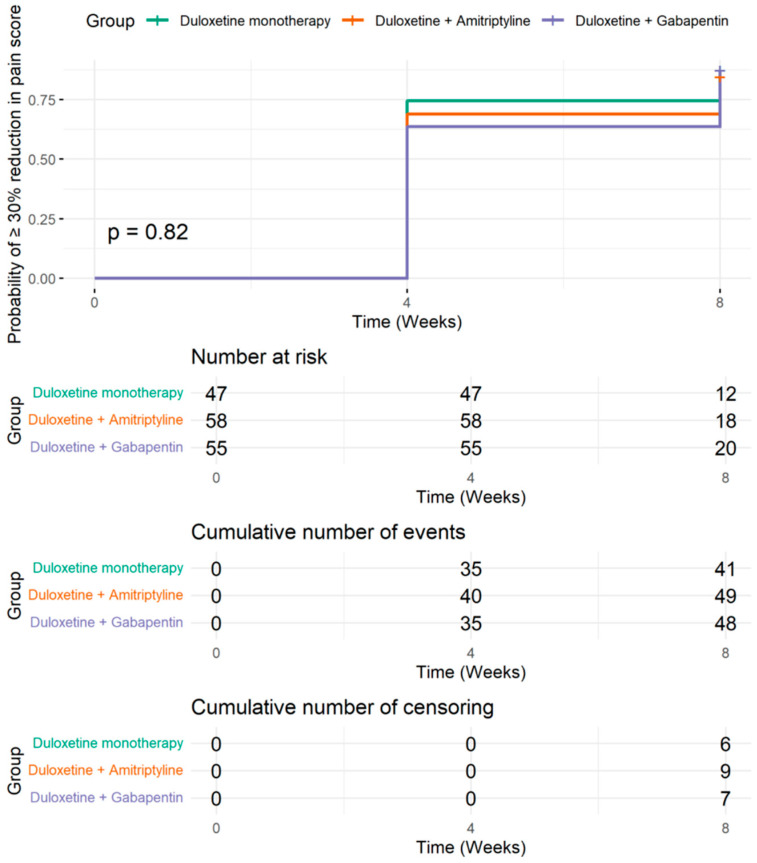
Time to achieving ≥30% reduction in average pain score among patients with moderate-to-severe chemotherapy-induced peripheral neuropathy, stratified by treatment group. Kaplan–Meier curves show the cumulative probability of achieving a ≥30% reduction.

Figure 2 illustrates the probability of achieving a clinically meaningful pain response (≥30% reduction in average pain score) over time among patients in the three treatment arms. By Week 4, approximately three-quarters of patients in each group had achieved this level of improvement, and by Week 8, cumulative responder rates exceeded 74% across all groups. The cumulative incidence of response was slightly higher in the duloxetine + amitriptyline group (84.5%) compared to duloxetine monotherapy (78.7%) and duloxetine + gabapentin (74.5%), consistent with proportions reported in Supplementary Table S3. However, the Kaplan–Meier analysis revealed no statistically significant difference in time to response between groups (log-rank *p* = 0.82), indicating that the onset of analgesic benefit occurred at a similar pace regardless of the treatment strategy Figure 2.

### 2.5. Predictors of Time to Achieving Clinically Meaningful Pain Reduction

Table 3 presents the results of Cox regression analyses examining predictors of time to achieving a clinically meaningful analgesic response (≥30% reduction in average pain score) over the 8-week follow-up. In the multiple regression model, patients who started treatment with chemotherapy achieved pain reduction significantly faster than those who did not (aHR = 2.11, 95% CI: 1.17–3.79, *p* = 0.013). In contrast, stage III disease was associated with a slower time to response (aHR = 0.46, 95% CI: 0.24–0.90, *p* = 0.022). Compared with platinum-based regimens, both Taxane-based chemotherapy (aHR = 0.52, 95% CI: 0.28–0.94, *p* = 0.032) and other chemotherapy classes (aHR = 0.41, 95% CI: 0.18–0.93, *p* = 0.033) were associated with a slower time to response. No statistically significant differences in time to response were observed between treatment groups, and other demographic or clinical variables were not significant predictors after adjustment.

### 2.6. Safety Profile and Treatment-Related Side Effects

Table 4 summarizes treatment-related adverse events reported during the 8-week study period. Insomnia occurred exclusively in the duloxetine monotherapy group and was reported by 12.8% of patients, a significantly higher frequency compared to the combination groups (*p* = 0.001). Gastrointestinal disturbances and dizziness with dry mouth were more commonly reported in the duloxetine + amitriptyline group (6.9% each), but these differences did not reach statistical significance. Dizziness and dry mouth were also reported by 8.6% of patients receiving amitriptyline, while 14.5% of patients in the duloxetine + gabapentin group experienced fatigue and dizziness. Overall, no serious adverse events were reported, and the combination regimens were generally well tolerated.

### 2.7. Subgroup Analyses

To further address potential effect modification by demographic characteristics, subgroup analyses stratified by age and sex were performed. Baseline characteristics across age and sex strata are presented in Supplementary Table S4, showing generally comparable distributions of treatment groups and clinical variables, with some expected differences in demographic and disease-related factors. The covariates included in the subgroup regression models were selected from variables demonstrating statistically significant differences between strata in Supplementary Table S4, while variables with extremely unbalanced distributions were excluded. This approach was adopted to ensure stable model estimates, given that stratification reduced the effective sample size within each subgroup. Stratified logistic and Cox regression analyses demonstrated consistent findings across subgroups, with no statistically significant differences in treatment effects between groups (Supplementary Table S5). Kaplan–Meier analyses similarly showed comparable probabilities of achieving ≥30% pain reduction across treatment groups within both age and sex strata (Supplementary Figures S1 and S2), with non-significant log-rank tests. Formal interaction analyses further confirmed the absence of statistically significant effect modification by sex or continuous age for either the logistic or Cox regression models (Supplementary Table S6). Overall, these analyses indicate that the treatment effect was consistent across age and sex subgroups.

## 3. Discussion

In this study of 160 patients with moderate-to-severe chemotherapy-induced peripheral neuropathy (CIPN), baseline imbalances were present across groups (e.g., co-existing disease prevalence, chemotherapy duration category, and radiation exposure). These differences could have influenced symptom trajectories and perceived response and should be considered when interpreting comparative effectiveness. The main study results for duloxetine monotherapy and duloxetine combined with either amitriptyline or gabapentin yielded clinically meaningful pain reductions over 8 weeks. All three regimens showed significant within-group improvements in pain severity domains. Improvements in patient-reported pain relief were observed in the duloxetine monotherapy and duloxetine + gabapentin arms, whereas the duloxetine + amitriptyline arm showed a more variable change in pain relief [1,2], although duloxetine monotherapy had the largest absolute reduction in average and current pain scores. This is consistent with previous RCTs and meta-analyses showing duloxetine’s superiority over a placebo but mixed results when compared with other agents or combinations [3].

The addition of amitriptyline to duloxetine significantly reduced the odds of inadequate analgesic response (adjusted OR = 0.23, *p* = 0.039). While amitriptyline is not specifically recommended in CIPN by the ASCO, its efficacy in diabetic and post-herpetic neuropathy suggests a benefit via the modulation of descending inhibitory pain pathways [4,5].

Conversely, duloxetine + gabapentin did not significantly outperform duloxetine alone. This aligns with the broader literature: gabapentin and pregabalin have demonstrated limited benefit yet better improvement at Week 8 in CIPN [6,7].

However, after multiplicity adjustment (Bonferroni), between-group differences between Weeks 4 and 8 were not statistically significant, indicating no clearly superior regimen.

In our study, all groups demonstrated similar responder rates (74.5–82.8%) by Week 8, defined as ≥30% reduction in average pain. These rates are higher than those reported in some duloxetine monotherapy trials (~59%) [8]. Given the absence of an absolute difference between groups after correction for multiple comparisons, duloxetine remains an evidence-supported first-line option for established painful CIPN [1]. In selected patients who do not respond adequately to duloxetine alone, individualized combination therapy may be considered with careful adverse-effect monitoring.

Regarding our multivariate analysis, the study revealed that co-treatment with amitriptyline, family history of neuropathy, chemotherapy as a first treatment, regression of cancer, and >6-month chemotherapy duration were associated with a greater likelihood of achieving sufficient pain relief. These results suggest a role for early intervention and patient-specific factors in optimizing response [9].

In contrast, patients with stage III or IV disease, Taxane-based chemotherapy, non-platinum chemotherapy, or prior surgery were more likely to exhibit inadequate response. These factors may reflect more severe neuronal damage or systemic inflammation, making pain less reversible [9,10].

Taken together, the present results suggest a benefit in pain intensity reduction, but the full clinical meaning of this improvement should be interpreted with caution.

Further results in our study showed that time-to-event analysis confirmed that ~75% of patients in all groups achieved a ≥30% pain reduction by Week 4, with no significant differences between treatments (log-rank *p* = 0.82). Starting treatment with chemotherapy was associated with faster response (aHR = 2.11, *p* = 0.013), reinforcing the potential benefit of early pharmacologic intervention [11].

Slower responses were seen in patients on Taxane-based or non-platinum regimens, and in those with stage III disease, reflecting neuropathy severity and the cumulative burden of neurotoxicity [12].

Among the reported adverse events, insomnia was significantly more common in the duloxetine monotherapy group (12.8%, *p* = 0.001), consistent with duloxetine’s activating profile as an SNRI. Mild GI effects and dizziness were more common in the amitriptyline group, and fatigue was most frequent in the gabapentin group. Overall, all treatments were well tolerated, with no serious adverse events reported [13].

The results of this study demonstrate that combination therapy with duloxetine plus either gabapentin or amitriptyline leads to possible pain reduction that can improve quality of life compared to duloxetine monotherapy; the implications for clinical practice could be significant. First, it could result in an update to clinical guidelines, providing clinicians with evidence-based support for using combination regimens in patients with refractory CIPN pain. Second, it may help standardize treatment strategies, thereby reducing the current variability driven by off-label use and insurance requirements.

Limitations:

The open-label design of clinical studies can introduce bias from patients and clinicians, despite its necessity. Future studies should consider using double-blind methods to reduce subjective bias in result assessments.

In early-phase clinical trials, the sample size may limit the generalizability of findings. Further multicenter trials are needed to confirm the observed results across multiple patient demographics and treatment regimens. The heterogeneity of CIPN etiology is impacted by chemotherapeutic drugs and patient variables, including pre-existing neuropathies and co-morbid disorders. Future studies should examine the subgroup analysis to identify if certain patient features indicate better response to combination medication.

The main clinical implication of this trial is therefore more modest: the results are broadly compatible with current guideline-based practice, which identifies **duloxetine** as the pharmacologic agent with the best-supported evidence for established painful CIPN, while the addition of gabapentin or amitriptyline may be considered only on an individualized basis when monotherapy is insufficient and tolerability has been carefully weighed.

## 4. Materials and Methods

### 4.1. Trial Design

This study is an open-label, phase-2, randomized, parallel-group, three-arm clinical trial conducted under a superiority framework, designed to determine whether duloxetine augmentation with gabapentin or amitriptyline provides greater improvement in chemotherapy-induced peripheral neuropathy (CIPN)-related pain than duloxetine monotherapy.

### 4.2. Allocation Ratio

**Figure 3:**
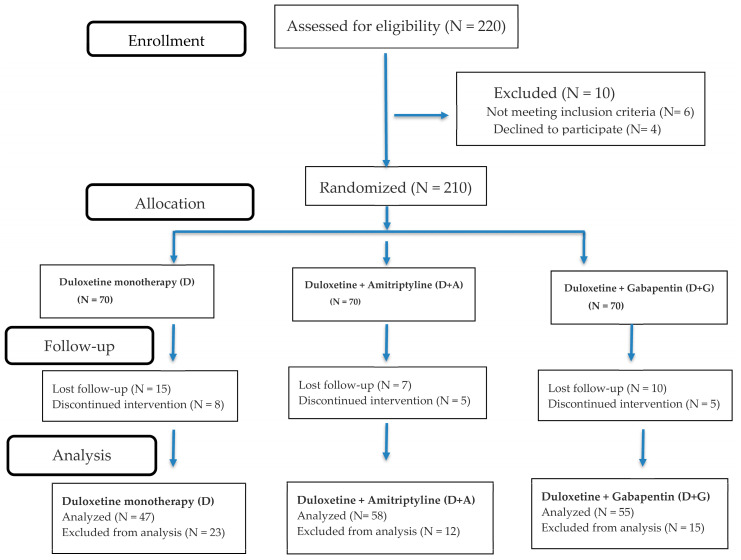
Consort flow chart adapted from research methods and reporting consort 2025 explanation and elaboration [14].

Eligible participants were randomized in a 1:1:1 allocation ratio to one of three treatment arms: duloxetine alone (Arm D) which represents the reference; duloxetine + amitriptyline (Arm D + A); or duloxetine + gabapentin (Arm D + G) (Figure 3).

### 4.3. Setting

The study was a single-center study which was conducted at the Oncology Department of Beni-Suef University Hospital, a tertiary-care teaching hospital located in Beni Suef, Egypt.

Participants were recruited from the oncology service (tertiary healthcare setting). The trial was conducted from October 2023 to July 2025. The ethical approval (Ref. No. FMBSUREC/03102023) to conduct the study was obtained from the Institutional Ethics Committee before starting the study.

### 4.4. Participants (Eligibility)

Patients must sign an informed consent form (ICF) voluntarily and be able to understand and comply with the requirements of the study, including being 18 to 75 years of age (including cut-offs) on the date of signing the informed consent form. Patients must receive treatment with a chemotherapy regimen known to induce CIPN. Patients must have ≥ Grade 1 sensory chemotherapy-induced peripheral neuropathy (CIPN) according to the NCI Common Toxicity. Criteria for Adverse Events (CTCAE) v.5.0 grading scale, Eastern Cooperative Oncology Group performance status (ECOG PS): 0–2, expected survival of ≥6 months.

### 4.5. Exclusion Criteria

History of allergic reactions attributed to compounds of similar chemical or biologic composition to duloxetine, amitriptyline and gabapentin; pregnant women; life expectancy less than 6 months; inability or unwillingness to comply with research protocols; the presence of active brain or meningeal metastases; uncontrolled closed-angle glaucoma; mental illness, epilepsy, mania, suicidal depression, dementia or alcohol or drug abuse that may have an impact on compliance with trial requirements; or the presence of co-morbid cardiovascular disease, including but not limited to:

(1) New York Heart Association (NYHA) criteria ≥ grade 2 heart failure;

(2) Severe/unstable angina pectoris;

(3) Myocardial infarction or cerebrovascular accident within 6 months prior to first dose;

(4) Atrial fibrillation and supraventricular or ventricular arrhythmias requiring treatment;

(5) Pre-existing symptomatic superior vena cava syndrome;

(6) Corrected QT interval (QTc) > 450 ms (men); QTc > 470 ms (women);

(7) Hypertensive disease not controlled by antihypertensive medication: systolic blood pressure ≥ 140 mmHg or diastolic blood pressure ≥ 90 mmHg.

### 4.6. Interventions (Treatment Regimens)

Participants were randomized to:

1. Duloxetine alone (Arm D): 30 mg once daily for 1 week, then 60 mg once daily.
2. Duloxetine + amitriptyline (Arm D + A): duloxetine as above, plus amitriptyline 10–25 mg at bedtime. Where duloxetine started first for two weeks, then add on amitriptyline.
3. Duloxetine + gabapentin (Arm D + G): duloxetine as above, plus gabapentin 300 mg at bedtime, with dose escalation as needed per response. Where duloxetine started first for two weeks, then add on gabapentin.
4. A key consideration in dosage regimen that we used in the trial is that amitriptyline and gabapentin were used within a response- and tolerability-guided titration protocol, rather than as fixed low-dose regimens. Amitriptyline was initiated at 10 or 25 mg/day and could be escalated up to 75 mg/day, while gabapentin was similarly titrated based on the clinical response and tolerability. This pragmatic strategy was chosen to reflect routine oncology practice, where escalation may be limited by sedation, dizziness, fatigue, anticholinergic effects, and overall treatment burden. However, because not all patients may have reached higher doses, the possibility of reduced observed efficacy due to incomplete titration should be acknowledged. This is particularly important in CIPN, where evidence for gabapentin and amitriptyline remains inconsistent, whereas duloxetine remains the only pharmacologic therapy supported by current ASCO guidance for established painful CIPN.
5. Concomitant and rescue analgesics: Concomitant and rescue analgesics were documented at baseline in the present study; the concomitant analgesics used for symptomatic pain management were tramadol as the opioid analgesic and paracetamol (acetaminophen) as the non-opioid analgesic. For each participant, analgesic exposure was categorized as opioid and/or non-opioid use at baseline, and these variables were incorporated into the prespecified regression models as potential covariates. Where available in the study records, the agent name, dose and frequency are summarized in Supplementary Table S7.

### 4.7. Outcomes and Follow-Up Schedule

Assessments were performed at baseline, Week 4, and Week 8.

The primary endpoint was the between-group difference in daily pain intensity measured using the Brief Pain Inventory—Short Form (BPI-SF) at the follow-up visits (Weeks 4 and 8) relative to baseline.

Secondary endpoints included the evaluation of a clinically meaningful pain response, defined a priori as a ≥30% reduction from baseline in the pain score, which represents a clinically relevant improvement in pain intensity. In addition, the time to achieving a ≥30% reduction in pain score during the 8-week follow-up period was analyzed to evaluate differences in the timing of clinically meaningful pain improvement across treatment groups.

### 4.8. Safety Monitoring

- Adverse effects were monitored throughout the follow-up and summarized by treatment group over the 8-week period.
- Baseline CIPN grading used NCI CTCAE v5.0.

### 4.9. Sample Size Calculation

The sample size was calculated in R (version 4.2.2) using the “pwr.chisq.test” function from the “pwr” package for a chi-square test comparing the proportion of responders (≥30% reduction in BPI-SF average pain score at Week 8) across three treatment groups. A medium effect size (Cohen’s w = 0.30) was assumed, corresponding to a moderate difference in responder rates between any two groups. For a 3 × 2 table (three treatment groups by responder status), the degrees of freedom were df = 2. With a two-sided α = 0.05 and 80% power, the required total sample size was N = 107.05 (≈108 patients), where N represents the total number of participants across all groups. To accommodate possible loss to follow-up and maintain adequate statistical power, we inflated this target by approximately 50%, aiming for at least 160 participants. The final analytic sample comprised 160 patients (47, 58, and 55 per group), exceeding the minimum required and ensuring the robustness of the primary categorical comparison.

### 4.10. Statistical Analysis

Data was analyzed using R (version 4.2.2; R Foundation for Statistical Computing). Baseline characteristics were summarized as mean ± standard deviation (SD) for continuous variables and frequency (percentage) for categorical variables. Between-group comparisons at baseline used one-way ANOVA for continuous variables and Pearson’s chi-square or Fisher’s exact tests for categorical variables, as appropriate.

Within-group changes in pain severity and pain relief from baseline to Weeks 4 and 8 were estimated using generalized estimating equations (GEEs) with an identity link and an exchangeable working correlation to account for repeated measures. Baseline was the reference time point, and results were expressed as mean change (MC) with 95% confidence intervals (CIs). Pairwise between-group comparisons at each time point were obtained from the GEE models and expressed as mean differences (MDs) with 95% confidence intervals. Bonferroni correction was applied separately for each outcome to account for the nine pairwise between-group comparisons across the three time points. Both unadjusted and covariate-adjusted GEE models were estimated; the adjusted models included baseline variables that showed imbalance between groups (co-existing disease, chemotherapy duration >6 months, and receipt of radiotherapy). Effect sizes and their 95% confidence intervals were emphasized in the interpretation of results, with adjusted *p*-values reported to account for multiple testing.

Responder status was defined a priori as achieving a ≥30% reduction from baseline in the average pain item of the BPI-SF. Between-group comparisons of responder proportions used chi-square tests; comparisons of the percent reduction in average pain used ANOVA.

Predictors of insufficient response in Week 8 (non-responders) were evaluated using binary logistic regression. All prespecified covariates were included in the multivariable model irrespective of their univariable *p*-values. The covariate set comprised treatment group, age, sex, BMI, opioid analgesic use, non-opioid analgesic use, family history of neuropathy, vitamin B12 supplementation, co-existing disease, cancer type, cancer status (progression, regression, stable), cancer stage (I–IV), metastasis, treatment initiated with chemotherapy, chemotherapy class (platinum, Taxane, others), total chemotherapy duration (<6 vs. >6 months), dose reduction due to side effects, prior surgery, and radiation therapy. Results were reported as odds ratios (ORs) and adjusted odds ratios (aORs) with 95% CIs.

Time-to-response (first achievement of ≥30% reduction in average pain) was analyzed using Kaplan–Meier estimates and compared across groups with the log-rank test. Predictors of time-to-response were assessed using Cox proportional hazards regression, including the same prespecified covariate set as above; results were expressed as hazard ratios (HRs) and adjusted hazard ratios (aHRs) with 95% CIs.

All tests were two-sided, and *p* < 0.05 was considered statistically significant.

## 5. Conclusions

In this randomized controlled trial, duloxetine monotherapy and its combination with amitriptyline or gabapentin had adjuvant reductions in CIPN-related pain. However, the addition of either agent did not provide superior analgesic impact compared with duloxetine alone. These findings encourage duloxetine as an evidence-based first-line treatment for painful CIPN, while a combination regimen may be considered on an individualized basis for patients showing inadequate response. Larger, multicenter trials are needed to confirm these results and further illustrate optimal treatment strategies.

## Figures and Tables

**Table 1:** Demographic, clinical, and treatment-related characteristics of patients with moderate-to-severe chemotherapy-induced peripheral neuropathy at baseline, stratified by treatment group (duloxetine monotherapy, duloxetine + amitriptyline, duloxetine + gabapentin).

Variables	Total (N = 160)	Duloxetine Monotherapy (N = 47)	Duloxetine + Amitriptyline (N = 58)	Duloxetine + Gabapentin (N = 55)	*p*
Age	48.9 ± 13.6	48.1 ± 14.1	49.8 ± 13.7	48.7 ± 13.2	0.801
Gender					
Female	99 (61.9)	33 (70.2)	34 (58.6)	32 (58.2)	0.375
Male	61 (38.1)	14 (29.8)	24 (41.4)	23 (41.8)	
BMI	28.3 ± 6.7	27.1 ± 7	28 ± 6.9	29.6 ± 6.1	0.151
Serum creatinine	0.8 ± 0.2	0.8 ± 0.2	0.8 ± 0.2	0.8 ± 0.1	0.841
Hb at baseline	11.7 ± 1.8	11.5 ± 1.9	11.9 ± 1.8	11.6 ± 1.7	0.414
Total leukocyte count	5.7 ± 3	5.7 ± 3.5	5.5 ± 2.3	6.1 ± 3.3	0.581
CTCEA neuropathy					
Grade 1	109 (68.1)	28 (59.6)	42 (72.4)	39 (70.9)	0.180
Grade 2	29 (18.1)	8 (17)	12 (20.7)	9 (16.4)	
Grade 3	22 (13.8)	11 (23.4)	4 (6.9)	7 (12.7)	
Opioid analgesic use	69 (43.1)	20 (42.6)	22 (37.9)	27 (49.1)	0.486
Non-opioid analgesic use	43 (26.9)	12 (25.5)	12 (20.7)	19 (34.5)	0.244
Family history	35 (21.9)	11 (23.4)	8 (13.8)	16 (29.1)	0.138
B12 use	128 (80)	37 (78.7)	48 (82.8)	43 (78.2)	0.804
Co-existing disease	32 (20)	5 (10.6)	18 (31)	9 (16.4)	0.024 *
Cancer type					
Breast	76 (47.5)	26 (55.3)	22 (37.9)	28 (50.9)	0.176
Colo-rectal	45 (28.1)	9 (19.1)	18 (31)	18 (32.7)	
Others	39 (24.4)	12 (25.5)	18 (31)	9 (16.4)	
Cancer status					
Progression	21 (13.1)	8 (17)	5 (8.6)	8 (14.5)	0.449
Regression	17 (10.6)	6 (12.8)	4 (6.9)	7 (12.7)	
Stable	122 (76.2)	33 (70.2)	49 (84.5)	40 (72.7)	
Time to progression					
<2 years	11 (6.9)	3 (6.4)	2 (3.4)	6 (10.9)	0.359
≥2 years	10 (6.2)	5 (10.6)	3 (5.2)	2 (3.6)	
Stable or regressed	139 (86.9)	39 (83)	53 (91.4)	47 (85.5)	
Stage					
Stage I	57 (35.6)	19 (40.4)	18 (31)	20 (36.4)	0.684
Stage II	40 (25)	12 (25.5)	18 (31)	10 (18.2)	
Stage III	53 (33.1)	13 (27.7)	18 (31)	22 (40)	
Stage IV	10 (6.2)	3 (6.4)	4 (6.9)	3 (5.5)	
Metastasis	49 (30.6)	13 (27.7)	21 (36.2)	15 (27.3)	0.513
Treatment start with chemotherapy	122 (76.2)	39 (83)	40 (69)	43 (78.2)	0.224
Chemotherapy class					
Platinum-based chemotherapy	76 (47.5)	23 (48.9)	27 (46.6)	26 (47.3)	0.982
Taxane-based chemotherapy (Taxol-dominant)	68 (42.5)	20 (42.6)	24 (41.4)	24 (43.6)	
Others	16 (10)	4 (8.5)	7 (12.1)	5 (9.1)	
Chemotherapy total duration					
<6 mo	35 (21.9)	6 (12.8)	11 (19)	18 (32.7)	0.042 *
>6 mo	125 (78.1)	41 (87.2)	47 (81)	37 (67.3)	
Dose reduction related to side effect	53 (33.1)	19 (40.4)	21 (36.2)	13 (23.6)	0.164
Surgery	113 (70.6)	29 (61.7)	41 (70.7)	43 (78.2)	0.190
Radiation	70 (43.8)	30 (63.8)	21 (36.2)	19 (34.5)	0.004 *

* *p*-value is significant, Values are presented as mean ± standard deviation (SD) for continuous variables and frequency (%) for categorical variables. Comparisons across treatment groups were conducted using one-way ANOVA for continuous variables and chi-square or Fisher’s exact tests for categorical variables. Bold *p*-values indicate statistically significant differences (*p* < 0.05). CTCEA = Common Terminology Criteria for Adverse Events; CIPN = chemotherapy-induced peripheral neuropathy; GIT = gastrointestinal tract; B12 = vitamin B12.

**Table 2:** Simple and multiple logistic regression analyses of predictors of insufficient response (<30% reduction in average pain score) in Week 8 among patients with moderate-to-severe chemotherapy-induced peripheral neuropathy.

Variable	OR (95% CI)	*p*	aOR (95% CI)	*p*
Group				
Duloxetine monotherapy	Reference		Reference	
Duloxetine + amitriptyline	0.77 (0.29, 2.07)	0.601	0.23 (0.05, 0.89)	0.039 *
Duloxetine + gabapentin	1.26 (0.50, 3.26)	0.620	0.65 (0.15, 2.77)	0.563
Age	0.98 (0.96, 1.01)	0.252	0.99 (0.94, 1.03)	0.627
Gender				
Female	Reference		Reference	
Male	1.37 (0.63, 2.96)	0.419	0.76 (0.08, 8.62)	0.821
BMI	0.95 (0.89, 1.00)	0.072	0.94 (0.86, 1.02)	0.154
Opioid analgesic use	0.56 (0.24, 1.22)	0.156	0.77 (0.21, 2.71)	0.691
Non-opioid analgesic use	0.43 (0.19, 0.96)	***0.037***	0.79 (0.22, 2.98)	0.718
Family history	0.55 (0.18, 1.45)	0.260	0.13 (0.02, 0.58)	0.014 *
B12 use	0.51 (0.22, 1.25)	0.126	0.66 (0.16, 2.75)	0.564
Co-existing disease	0.20 (0.03, 0.71)	***0.034***	0.16 (0.02, 1.03)	0.075
Cancer type				
Breast	Reference		Reference	
Colo-rectal	1.48 (0.61, 3.53)	0.378	0.92 (0.04, 17.87)	0.956
Others	0.89 (0.31, 2.34)	0.818	2.83 (0.25, 33.81)	0.398
Cancer status				
Progression	Reference		Reference	
Regression	0.80 (0.10, 5.45)	0.819	0.01 (0.00, 0.24)	0.009 *
Stable	1.87 (0.58, 8.39)	0.342	0.16 (0.02, 1.39)	0.099
Stage				
Stage I	Reference		Reference	
Stage II	2.01 (0.77, 5.37)	0.153	3.41 (0.56, 22.90)	0.188
Stage III	1.09 (0.41, 2.91)	0.857	10.03 (1.51, 83.33)	0.022 *
Stage IV	1.18 (0.16, 5.63)	0.852	52.75 (1.49, 2266.13)	0.030 *
Metastasis	0.52 (0.19, 1.23)	0.157	0.29 (0.04, 1.58)	0.172
Treatment start with chemotherapy	0.24 (0.10, 0.54)	***<0.001***	0.05 (0.01, 0.23)	<0.001 *
Chemotherapy class				
Platinum-based	Reference		Reference	
Taxane-based (Taxol-dominant)	0.82 (0.36, 1.84)	0.632	9.05 (1.38, 79.01)	***0.032*** *
Others	1.16 (0.29, 3.83)	0.820	24.61 (1.92, 428.76)	0.019 *
Chemotherapy total duration				
<6 months	Reference		Reference	
>6 months	0.24 (0.10, 0.55)	***<0.001***	0.09 (0.01, 0.47)	*0.008* *
Dose reduction related to side effect	0.96 (0.41, 2.11)	0.914	0.55 (0.15, 1.91)	0.363
Surgery	1.20 (0.53, 2.94)	0.675	7.92 (1.20, 65.40)	***0.041*** *
Radiation	0.38 (0.16, 0.86)	***0.025***	0.45 (0.08, 2.38)	0.352

* *p* values < 0.05 are considered statistically significant and are presented in bold italic. Response was defined as a ≥30% reduction in average pain score from baseline at Week 8 based on the Brief Pain Inventory—Short Form (BPI-SF).

**Table 3:** Simple and multiple Cox regression analyses of predictors of time to achieving ≥30% reduction in average pain score among patients with moderate-to-severe chemotherapy-induced peripheral neuropathy.

Variable	HR (95% CI)	*p*	aHR (95% CI)	*p*
Group				
Duloxetine monotherapy	Reference		Reference	
Duloxetine + amitriptyline	0.89 (0.59, 1.35)	0.584	1.16 (0.72, 1.88)	0.537
Duloxetine + gabapentin	0.88 (0.58, 1.33)	0.534	0.92 (0.56, 1.51)	0.745
Age	1.01 (1.00, 1.03)	***0.033***	1.01 (1.00, 1.03)	0.187
Gender				
Female	Reference		Reference	
Male	0.86 (0.61, 1.21)	0.387	0.79 (0.41, 1.51)	0.478
BMI	1.02 (0.99, 1.04)	0.173	1.01 (0.98, 1.04)	0.593
Opioid analgesic use	1.21 (0.86, 1.69)	0.276	1.07 (0.70, 1.61)	0.765
Non-opioid analgesic use	0.84 (0.58, 1.22)	0.364	0.64 (0.40, 1.04)	0.070
Family history	1.24 (0.84, 1.83)	0.286	1.44 (0.94, 2.22)	0.097
B12 use	0.79 (0.52, 1.20)	0.278	0.85 (0.49, 1.49)	0.579
Co-existing disease	1.46 (0.98, 2.18)	0.066	1.63 (0.94, 2.83)	0.082
Cancer type				
Breast	Reference		Reference	
Colo-rectal	0.88 (0.59, 1.32)	0.549	0.89 (0.35, 2.25)	0.812
Others	1.08 (0.72, 1.63)	0.702	0.71 (0.35, 1.45)	0.348
Cancer status				
Progression	Reference		Reference	
Regression	0.84 (0.43, 1.64)	0.605	1.77 (0.71, 4.38)	0.221
Stable	0.62 (0.38, 1.00)	0.050	0.97 (0.53, 1.76)	0.911
Stage				
Stage I	Reference		Reference	
Stage II	0.78 (0.50, 1.21)	0.262	0.63 (0.32, 1.23)	0.179
Stage III	0.87 (0.58, 1.30)	0.486	0.46 (0.24, 0.90)	0.022 *
Stage IV	1.59 (0.81, 3.15)	0.180	0.80 (0.28, 2.26)	0.671
Metastasis	1.29 (0.91, 1.85)	0.156	1.35 (0.81, 2.24)	0.247
Treatment start with chemotherapy	1.39 (0.92, 2.08)	0.114	2.11 (1.17, 3.79)	0.013 *
Chemotherapy class				
Platinum-based	Reference		Reference	
Taxane-based (Taxol-dominant)	0.85 (0.60, 1.21)	0.375	0.52 (0.28, 0.94)	0.032 *
Others	0.69 (0.37, 1.28)	0.236	0.41 (0.18, 0.93)	0.033 *
Chemotherapy total duration				
<6 months	Reference		Reference	
>6 months	1.42 (0.93, 2.18)	0.106	1.15 (0.64, 2.08)	0.636
Dose reduction related to side effect	1.02 (0.71, 1.46)	0.912	0.94 (0.60, 1.46)	0.775
Surgery	0.89 (0.62, 1.29)	0.553	0.74 (0.42, 1.30)	0.292
Radiation	1.26 (0.90, 1.77)	0.173	1.04 (0.61, 1.78)	0.892

Values are presented as hazard ratio (HR) and adjusted hazard ratio (aHR) with 95% confidence intervals (CIs). HRs are derived from simple Cox regression models, and aHRs from multiple Cox regression models including all variables shown. * *p* values < 0.05 are considered statistically significant and are presented in bold italic. Response was defined as achieving a ≥30% reduction in average pain score from baseline based on the Brief Pain Inventory—Short Form (BPI-SF).

**Table 4:** Frequency of treatment-related adverse events reported over 8 weeks in patients with moderate-to-severe chemotherapy-induced peripheral neuropathy, by treatment group.

Variables	Total	Duloxetine Monotherapy	Duloxetine + Amitriptyline	Duloxetine + Gabapentin	*p*
Duloxetine-related side effects					
Insomnia	6 (3.8)	6 (12.8)	0 (0)	0 (0)	0.001 *
GIT disturbance	5 (3.1)	1 (2.1)	4 (6.9)	0 (0)	0.098
Dizziness and dry mouth	5 (3.1)	1 (2.1)	4 (6.9)	0 (0)	0.098
Amitriptyline-related side effects					
Dizziness and dry mouth			5 (8.6)		
Gabapentin-related side effects					
Fatigue and dizziness				8 (14.5)	

Values are presented as frequency (%). Comparisons across treatment groups were performed using chi-square or Fisher’s exact test as appropriate. * *p*-values < 0.05 were considered statistically significant and are shown in bold. Side effects were categorized according to drug-specific profiles and clinical assessment. GIT = gastrointestinal tract.

## Data Availability

The original contributions presented in this study are included in the article/Supplementary Materials. Further inquiries can be directed to the corresponding author.

## Supplementary Materials

The following supporting information can be downloaded at: https://www.mdpi.com/article/10.3390/ph19040553/s1. Table S1. Within-Group Changes in Pain Severity and Pain Relief Compared to Baseline Over 8 Weeks in Patients with Moderate-to-Severe Chemotherapy-Induced Peripheral Neuropathy, by Treatment Arm. Table S2. Pairwise Comparisons of Pain Scores and Pain Relief Between Treatment Groups at Each Time Point in Patients with Moderate-to-Severe Chemotherapy-Induced Peripheral Neuropathy over 8 weeks. Table S3. Percentage Reduction in Average Pain Scores and Proportion of Responders at Weeks 4 and 8 in Patients with Moderate-to-Severe Chemotherapy-Induced Peripheral Neuropathy, by Treatment Group. Table S4. Baseline characteristics of the study population stratified by age group and sex. Table S5. Subgroup analyses of treatment effect stratified by age and sex using multivariable logistic and Cox regression models. Table S6. Interaction analyses evaluating effect modification by sex and continuous age on the association between treatment group and pain reduction outcomes. Table S7. Concomitant and rescue analgesics used during the study. Figure S1. Kaplan–Meier curves of time to ≥30% pain reduction stratified by sex. Figure S2. Kaplan–Meier curves of time to ≥30% pain reduction stratified by age group.
