# Supplementary material for: Efficacy and Safety of Duloxetine with Gabapentin or Amitriptyline Versus Duloxetine Monotherapy in Chemotherapy-Induced Peripheral Neuropathy: Randomized Controlled Trial
Source: Pharmaceuticals (Basel). 2026 Mar 30;19(4):553. doi: 10.3390/ph19040553 (PMC13118665; doi:10.3390/ph19040553)
Supplement: Supplementary file 1 [file pharmaceuticals-19-00553-s001.zip › pharmaceuticals-4170028-supplementary.pdf]

Table S1. Within-Group Changes in Pain Severity and Pain Relief Compared to Baseline Over 8 Weeks in Patients with Moderate to Severe Chemotherapy-Induced Peripheral Neuropathy, by Treatment Arm

Variable	Duloxetine monotherapy			Duloxetine + Amitriptyline			Duloxetine + Gabapentin		
	**M ± SD**	**MC (95% CI)**	**p**	**M ± SD**	**MC (95% CI)**	**p**	**M ± SD**	**MC (95% CI)**	**p**
**Worst pain in the last 24 hours**									
Baseline	4 ± 2.5	Reference		3.1 ± 1.9	Reference		3.4 ± 2.1	Reference	
Week 4	2.5 ± 1.4	-1.49 (-2.31, -0.67)	***<0.001***	2.2 ± 1.2	-0.90 (-1.47, -0.33)	***0.002***	2.5 ± 1.4	-0.91 (-1.57, -0.25)	***0.007***
Week 8	1.7 ± 1.5	-2.23 (-3.06, -1.41)	***<0.001***	1.4 ± 1.3	-1.69 (-2.28, -1.10)	***<0.001***	1.7 ± 1.1	-1.71 (-2.33, -1.09)	***<0.001***
**Least pain in the last 24 hours**									
Baseline	3.3 ± 1.1	Reference		3 ± 1.6	Reference		3 ± 1.2	Reference	
Week 4	1.8 ± 0.9	-1.47 (-1.88, -1.06)	***<0.001***	1.8 ± 1.1	-1.19 (-1.69, -0.69)	***<0.001***	2.1 ± 1.2	-0.84 (-1.27, -0.40)	***<0.001***
Week 8	1.6 ± 0.9	-1.66 (-2.08, -1.24)	***<0.001***	1.2 ± 1	-1.81 (-2.30, -1.32)	***<0.001***	1.8 ± 1.3	-1.18 (-1.63, -0.73)	***<0.001***
**Average pain**									
Baseline	3.3 ± 1.7	Reference		2.9 ± 1.6	Reference		3.2 ± 1.8	Reference	
Week 4	2 ± 1.2	-1.30 (-1.89, -0.71)	***<0.001***	1.9 ± 1.2	-1.03 (-1.55, -0.52)	***<0.001***	2.2 ± 1.3	-1.02 (-1.60, -0.43)	***<0.001***
Week 8	1.6 ± 1.1	-1.64 (-2.22, -1.06)	***<0.001***	1.3 ± 1.1	-1.62 (-2.12, -1.12)	***<0.001***	1.8 ± 1.3	-1.44 (-2.02, -0.86)	***<0.001***
**Pain right now**									
Baseline	3.5 ± 2.2	Reference		3 ± 1.5	Reference		3.2 ± 1.6	Reference	
Week 4	2 ± 1	-1.47 (-2.14, -0.79)	***<0.001***	2.1 ± 1.1	-0.88 (-1.36, -0.40)	***<0.001***	2.1 ± 1	-1.16 (-1.66, -0.67)	***<0.001***
Week 8	1.6 ± 1.1	-1.85 (-2.54, -1.16)	***<0.001***	1.3 ± 1.1	-1.71 (-2.18, -1.23)	***<0.001***	1.6 ± 1.2	-1.62 (-2.14, -1.09)	***<0.001***
**Pain relief in the last 24 hours**									
Baseline	42.8 ± 19	Reference		34 ± 17.9	Reference		34 ± 15.6	Reference	
Week 4	27.2 ± 13.3	-15.53 (-22.08, -8.98)	***<0.001***	29.5 ± 18.4	-4.48 (-11.02, 2.06)	0.179	27.5 ± 19.1	-6.55 (-13.00, -0.10)	***0.047***
Week 8	28.5 ± 26.9	-14.26 (-23.57, -4.94)	***0.003***	28.6 ± 27.4	-5.34 (-13.68, 2.99)	0.209	26.2 ± 25.1	-7.82 (-15.56, -0.07)	***0.048***

Values are presented as mean ± standard deviation (M ± SD) at each time point. MC: mean change from baseline; CI: confidence interval. Estimates were obtained using generalized estimating equations (GEE) for each treatment group separately, with baseline serving as the reference time point. Pain scores were measured using the Brief Pain Inventory – Short Form (BPI-SF), and pain relief was reported as a percentage over the past 24 hours. Bold p-values indicate statistically significant change from baseline ($p < 0.05$).

Table S2. Pairwise Between-Group Comparisons of Pain Intensity and Pain Relief at Each Time Point Using Unadjusted and Covariate-Adjusted Generalized Estimating Equation Models in Patients with Moderate to Severe Chemotherapy-Induced Peripheral Neuropathy Over 8 Weeks.

Variable	Comparisons and covariates	Unadjusted GEE			Adjusted GEE		
		MD (95% CI)	p	Adjusted p	MD (95% CI)	p	Adjusted p
**Worst pain in the last 24 hours**							
Baseline	D vs (D+A)	0.87 (0.01, 1.73)	***0.048***	0.431	0.87 (-0.00, 1.75)	0.051	0.455
	D vs (D+G)	0.56 (-0.35, 1.46)	0.227	1.000	0.55 (-0.40, 1.50)	0.253	1.000
	(D+A) vs (D+G)	-0.31 (-1.05, 0.42)	0.404	1.000	-0.32 (-1.06, 0.42)	0.393	1.000
Week 4	D vs (D+A)	0.28 (-0.23, 0.79)	0.284	1.000	0.30 (-0.25, 0.84)	0.292	1.000
	D vs (D+G)	-0.02 (-0.57, 0.52)	0.934	1.000	-0.03 (-0.60, 0.55)	0.923	1.000
	(D+A) vs (D+G)	-0.30 (-0.77, 0.17)	0.210	1.000	-0.32 (-0.81, 0.16)	0.190	1.000
Week 8	D vs (D+A)	0.33 (-0.21, 0.86)	0.229	1.000	0.34 (-0.22, 0.90)	0.233	1.000
	D vs (D+G)	0.03 (-0.46, 0.53)	0.898	1.000	0.03 (-0.48, 0.54)	0.917	1.000
	(D+A) vs (D+G)	-0.29 (-0.73, 0.14)	0.186	1.000	-0.31 (-0.76, 0.14)	0.171	1.000
Co-existing disease					0.01 (-0.37, 0.38)	0.976	
Chemotherapy duration >6mo					0.20 (-0.16, 0.56)	0.272	
Radiation					-0.12 (-0.46, 0.22)	0.498	
**Least pain in the last 24 hours**							
Baseline	D vs (D+A)	0.24 (-0.28, 0.76)	0.370	1.000	0.24 (-0.29, 0.78)	0.366	1.000
	D vs (D+G)	0.29 (-0.16, 0.74)	0.201	1.000	0.29 (-0.18, 0.76)	0.227	1.000
	(D+A) vs (D+G)	0.05 (-0.46, 0.57)	0.838	1.000	0.05 (-0.47, 0.56)	0.863	1.000
Week 4	D vs (D+A)	-0.04 (-0.43, 0.35)	0.839	1.000	-0.05 (-0.47, 0.37)	0.823	1.000
	D vs (D+G)	-0.34 (-0.74, 0.06)	0.096	0.867	-0.34 (-0.76, 0.08)	0.113	1.000
	(D+A) vs (D+G)	-0.30 (-0.72, 0.12)	0.166	1.000	-0.29 (-0.72, 0.14)	0.181	1.000
Week 8	D vs (D+A)	0.39 (0.01, 0.76)	***0.042***	0.379	0.39 (-0.01, 0.79)	0.053	0.479
	D vs (D+G)	-0.19 (-0.61, 0.24)	0.391	1.000	-0.19 (-0.62, 0.25)	0.396	1.000
	(D+A) vs (D+G)	-0.57 (-1.00, -0.15)	***0.008***	0.068	-0.58 (-1.00, -0.15)	***0.008***	0.068
Co-existing disease					-0.03 (-0.30, 0.24)	0.847	
Chemotherapy duration >6mo					0.11 (-0.16, 0.37)	0.440	
Radiation					-0.07 (-0.31, 0.17)	0.555	
**Average pain**							
Baseline	D vs (D+A)	0.32 (-0.32, 0.97)	0.322	1.000	0.35 (-0.30, 0.99)	0.293	1.000
	D vs (D+G)	0.06 (-0.62, 0.74)	0.873	1.000	0.09 (-0.62, 0.81)	0.798	1.000
	(D+A) vs (D+G)	-0.27 (-0.90, 0.36)	0.402	1.000	-0.25 (-0.89, 0.38)	0.435	1.000
Week 4	D vs (D+A)	0.06 (-0.39, 0.51)	0.789	1.000	0.08 (-0.40, 0.57)	0.735	1.000
	D vs (D+G)	-0.22 (-0.71, 0.26)	0.363	1.000	-0.19 (-0.69, 0.32)	0.470	1.000
	(D+A) vs (D+G)	-0.29 (-0.75, 0.17)	0.224	1.000	-0.27 (-0.74, 0.20)	0.263	1.000
Week 8	D vs (D+A)	0.31 (-0.12, 0.73)	0.154	1.000	0.34 (-0.10, 0.78)	0.128	1.000
	D vs (D+G)	-0.15 (-0.61, 0.32)	0.534	1.000	-0.11 (-0.58, 0.36)	0.648	1.000
	(D+A) vs (D+G)	-0.45 (-0.89, -0.01)	***0.043***	0.390	-0.45 (-0.89, -0.00)	***0.048***	0.433
Co-existing disease					-0.03 (-0.32, 0.27)	0.853	
Chemotherapy duration >6mo					0.06 (-0.26, 0.39)	0.696	
Radiation					-0.18 (-0.46, 0.10)	0.208	
**Pain right now**							
Baseline	D vs (D+A)	0.49 (-0.24, 1.21)	0.191	1.000	0.56 (-0.18, 1.31)	0.137	1.000

	D vs (D+G)	0.25 (-0.50, 1.00)	0.512	1.000	0.33 (-0.46, 1.12)	0.410	1.000
	(D+A) vs (D+G)	-0.24 (-0.81, 0.34)	0.422	1.000	-0.23 (-0.83, 0.36)	0.445	1.000
	D vs (D+A)	-0.10 (-0.50, 0.30)	0.610	1.000	-0.03 (-0.46, 0.41)	0.901	1.000
Week 4	D vs (D+G)	-0.05 (-0.43, 0.32)	0.774	1.000	0.03 (-0.37, 0.42)	0.891	1.000
	(D+A) vs (D+G)	0.05 (-0.33, 0.43)	0.800	1.000	0.06 (-0.34, 0.45)	0.784	1.000
	D vs (D+A)	0.34 (-0.08, 0.76)	0.112	1.000	0.41 (-0.02, 0.85)	0.060	0.536
Week 8	D vs (D+G)	0.02 (-0.43, 0.46)	0.940	1.000	0.10 (-0.33, 0.53)	0.650	1.000
	(D+A) vs (D+G)	-0.32 (-0.74, 0.09)	0.127	1.000	-0.32 (-0.72, 0.09)	0.129	1.000
Co-existing disease					0.05 (-0.23, 0.33)	0.716	
Chemotherapy duration >6mo					-0.05 (-0.38, 0.28)	0.765	
Radiation					-0.24 (-0.50, 0.03)	0.077	
**Pain relief in the last 24 hours**							
	D vs (D+A)	8.80 (1.74, 15.86)	***0.015***	0.132	6.98 (-0.40, 14.37)	0.064	0.575
Baseline	D vs (D+G)	8.77 (2.01, 15.52)	***0.011***	0.100	7.01 (0.09, 13.93)	***0.047***	0.425
	(D+A) vs (D+G)	-0.03 (-6.17, 6.10)	0.991	1.000	0.02 (-6.17, 6.21)	0.994	1.000
	D vs (D+A)	-2.25 (-8.28, 3.78)	0.464	1.000	-4.51 (-10.91, 1.89)	0.166	1.000
Week 4	D vs (D+G)	-0.22 (-6.49, 6.05)	0.945	1.000	-1.98 (-8.52, 4.56)	0.553	1.000
	(D+A) vs (D+G)	2.03 (-4.84, 8.90)	0.562	1.000	2.53 (-4.24, 9.31)	0.462	1.000
	D vs (D+A)	-0.11 (-10.47, 10.25)	0.983	1.000	-2.53 (-13.28, 8.21)	0.643	1.000
Week 8	D vs (D+G)	2.33 (-7.77, 12.42)	0.651	1.000	0.57 (-10.10, 11.24)	0.916	1.000
	(D+A) vs (D+G)	2.44 (-7.18, 12.06)	0.619	1.000	3.11 (-6.45, 12.66)	0.524	1.000
Co-existing disease					-4.31 (-8.99, 0.38)	0.072	
Chemotherapy duration >6mo					1.66 (-3.11, 6.43)	0.495	
Radiation					4.03 (-0.26, 8.31)	0.066	

Values represent mean differences (MD) with 95% confidence intervals (CI) between treatment groups at each time point. Estimates were obtained using generalized estimating equations (GEE) with an identity link and an exchangeable working correlation to account for repeated measures within subjects. Both unadjusted and covariate-adjusted GEE models are presented. The adjusted models included baseline variables that showed imbalance between groups in Table 1 (co-existing disease, chemotherapy duration >6 months, and receipt of radiotherapy). Pairwise comparisons were performed between duloxetine monotherapy (D), duloxetine + amitriptyline (D+A), and duloxetine + gabapentin (D+G) at Baseline, Week 4, and Week 8. Bonferroni correction was applied separately for each outcome variable to the nine pairwise comparisons across the three time points. Pain intensity was measured using Pain Inventory–Short Form (BPI-SF)for worst pain, least pain, average pain, and pain right now during the past 24 hours. Pain relief represents the percentage of pain relief experienced during the past 24 hours. Bold p-values indicate nominal statistical significance before adjustment ($p < 0.05$)..

Table S3. Percentage Reduction in Average Pain Scores and Proportion of Responders at Weeks 4 and 8 in Patients with Moderate to Severe Chemotherapy-Induced Peripheral Neuropathy, by Treatment Group

Variables	Total	Duloxetine monotherapy	Duloxetine + Amitriptyline	Duloxetine + Gabapentin	p
Percentage reduction in average pain scores from baseline (%)					
Week 4	21.6 ± 67.5	35.8 ± 32.2	10.5 ± 83.6	21.2 ± 69.6	0.161
Week 8	36.4 ± 65.6	39.9 ± 46.1	36.7 ± 84.6	33 ± 57.1	0.868
Responders (achieving ≥30% reduction in pain score)					
Week 4	110 (68.8)	35 (74.5)	40 (69)	35 (63.6)	0.500
Week 8	126 (78.8)	37 (78.7)	48 (82.8)	41 (74.5)	0.566

Values are presented as mean ± standard deviation (M ± SD) for percentage reduction in average pain scores and as frequency (%) for responder rates. Responders were defined as patients who achieved a ≥30% reduction in average pain score from baseline based on the Brief Pain Inventory – Short Form (BPI-SF). Comparisons across treatment groups were performed using ANOVA for continuous outcomes and chi-square tests for categorical outcomes. No statistically significant differences were observed across groups at either time point ($p > 0.05$).

Table S4. Baseline characteristics of the study population stratified by age group and sex

**Variables**	**Age ≤ 51 N = 87**	**Age > 51 N = 73**	**p**	**Female N = 99**	**Male N = 61**	**p**
Group						
Duloxetine monotherapy	29 (33.3)	18 (24.7)	0.389	33 (33.3)	14 (23)	0.375
Duloxetine + Amitriptyline	28 (32.2)	30 (41.1)		34 (34.3)	24 (39.3)	
Duloxetine + Gabapentin	30 (34.5)	25 (34.2)		32 (32.3)	23 (37.7)	
Age				47.2 ± 13.1	51.8 ± 14	***0.042***
Gender						
Female	58 (66.7)	41 (56.2)	0.231			
Male	29 (33.3)	32 (43.8)				
BMI	27.3 ± 5.3	29.4 ± 8	0.050	29.9 ± 6.2	25.6 ± 6.6	***<0.001***
Serum Creatinine	0.8 ± 0.2	0.8 ± 0.2	***0.026***	0.8 ± 0.2	0.9 ± 0.2	***0.003***
Hb at baseline	12.1 ± 1.5	11.1 ± 2	***<0.001***	11.6 ± 1.5	11.9 ± 2.2	0.341
Total Leukocyte Count	5.5 ± 3.1	6 ± 2.9	0.281	5.5 ± 3.3	6.1 ± 2.4	0.242
CTCEA neuropathy						
Grade 1	62 (71.3)	47 (64.4)	0.588	64 (64.6)	45 (73.8)	0.116
Grade 2	15 (17.2)	14 (19.2)		17 (17.2)	12 (19.7)	
Grade 3	10 (11.5)	12 (16.4)		18 (18.2)	4 (6.6)	
Opioid analgesic use	36 (41.4)	33 (45.2)	0.744	34 (34.3)	35 (57.4)	***0.007***
Non opioid analgesic use	64 (73.6)	53 (72.6)	1.000	77 (77.8)	40 (65.6)	0.132
Family history	25 (28.7)	10 (13.7)	***0.036***	24 (24.2)	11 (18)	0.468
B12 use	74 (85.1)	54 (74)	0.122	88 (88.9)	40 (65.6)	***<0.001***
Co-existing disease	5 (5.7)	27 (37)	***<0.001***	17 (17.2)	15 (24.6)	0.349
Cancer type						
breast	46 (52.9)	30 (41.1)	0.136	76 (76.8)	0 (0)	***<0.001***
Colo-rectal	25 (28.7)	20 (27.4)		5 (5.1)	40 (65.6)	
others	16 (18.4)	23 (31.5)		18 (18.2)	21 (34.4)	
Cancer status						
progression	11 (12.6)	10 (13.7)	0.662	16 (16.2)	5 (8.2)	***0.043***
regression	11 (12.6)	6 (8.2)		14 (14.1)	3 (4.9)	
stable	65 (74.7)	57 (78.1)		69 (69.7)	53 (86.9)	
time to progression						
< 2 years	3 (3.4)	8 (11)	0.056	6 (6.1)	5 (8.2)	0.022
≥2 years	8 (9.2)	2 (2.7)		10 (10.1)	0 (0)	
Stable or regressed	76 (87.4)	63 (86.3)		83 (83.8)	56 (91.8)	
Stage						
Stage I	32 (36.8)	25 (34.2)	0.293	36 (36.4)	21 (34.4)	0.426
Stage II	25 (28.7)	15 (20.5)		21 (21.2)	19 (31.1)	
Stage III	27 (31)	26 (35.6)		34 (34.3)	19 (31.1)	
Stage IV	3 (3.4)	7 (9.6)		8 (8.1)	2 (3.3)	
Metastasis	23 (26.4)	26 (35.6)	0.279	28 (28.3)	21 (34.4)	0.521
Treatment start with chemotherapy	68 (78.2)	54 (74)	0.665	79 (79.8)	43 (70.5)	0.249
Chemotherapy class						
Platinum-Based Chemotherapy	40 (46)	36 (49.3)	0.506	31 (31.3)	45 (73.8)	***<0.001***

Taxane-Based Chemotherapy (Taxol-Dominant)	40 (46)	28 (38.4)		64 (64.6)	4 (6.6)	
Others	7 (8)	9 (12.3)		4 (4)	12 (19.7)	
chemotherapy total Duration						
<6mo	27 (31)	8 (11)	***0.004***	21 (21.2)	14 (23)	0.951
>6mo	60 (69)	65 (89)		78 (78.8)	47 (77)	
Dose reduction related to side effect	25 (28.7)	28 (38.4)	0.263	37 (37.4)	16 (26.2)	0.200
Surgery	62 (71.3)	51 (69.9)	0.984	66 (66.7)	47 (77)	0.222
Radiation	37 (42.5)	33 (45.2)	0.857	53 (53.5)	17 (27.9)	***0.003***

Values are presented as n (%) for categorical variables and mean ± standard deviation (SD) for continuous variables. Comparisons between strata were performed using the Chi-square or Fisher's exact test for categorical variables and the independent samples t-test for continuous variables. Age groups were defined according to the median age of the study population (51 years). Variables showing statistically significant differences between strata were considered as candidate covariates for the subgroup multivariable models, while variables with extremely unbalanced distributions or rare occurrences were excluded to ensure stable estimates after stratification. BMI: Body mass index; Hb: Hemoglobin.

Table S5. Subgroup analyses of treatment effect stratified by age and sex using multivariable logistic and Cox regression models

**Subgroup Analysis by Sex**								
	**Sex subgroup: Female**				**Sex subgroup: Male**			
**term**	**Logistic regression**		**Cox regression**		**Logistic regression**		**Cox regression**	
	**aOR (95% CI)**	**p**	**aHR (95% CI)**	**p**	**aOR (95% CI)**	**p**	**aHR (95% CI)**	**p**
Group								
Duloxetine monotherapy	Reference		Reference		Reference		Reference	
Duloxetine + Amitriptyline	0.55 (0.14, 2.00)	0.371	1.09 (0.63, 1.88)	0.766	0.32 (0.01, 6.16)	0.444	0.78 (0.29, 2.07)	0.614
Duloxetine + Gabapentin	0.63 (0.14, 2.62)	0.533	1.21 (0.67, 2.20)	0.530	0.81 (0.07, 12.64)	0.868	1.37 (0.45, 4.16)	0.583
Age	0.98 (0.94, 1.03)	0.531	1.00 (0.98, 1.02)	0.851	1.01 (0.95, 1.08)	0.638	1.03 (1.00, 1.05)	***0.026***
BMI	0.95 (0.86, 1.05)	0.347	1.01 (0.97, 1.05)	0.791	0.93 (0.79, 1.07)	0.298	1.02 (0.96, 1.09)	0.451
Serum Creatinine	3.01 (0.07, 129.16)	0.560	1.62 (0.35, 7.54)	0.538	0.02 (0.00, 4.86)	0.171	1.74 (0.19, 15.69)	0.623
Opioid use	1.06 (0.31, 3.41)	0.918	1.06 (0.65, 1.72)	0.808	0.49 (0.08, 2.54)	0.400	1.13 (0.55, 2.32)	0.747
B12 use	0.60 (0.13, 3.44)	0.537	0.70 (0.34, 1.42)	0.321	0.32 (0.06, 1.70)	0.185	0.94 (0.44, 2.02)	0.876
Radiation	0.78 (0.24, 2.58)	0.679	0.98 (0.61, 1.57)	0.934	0.06 (0.00, 1.09)	0.118	2.59 (0.99, 6.77)	0.053
**Subgroup Analysis by Age**								
	**Age subgroup: ≤ 51**				**Age subgroup: > 51**			
**term**	**Logistic regression**		**Cox regression**		**Logistic regression**		**Cox regression**	
	**aOR (95% CI)**	**p**	**aHR (95% CI)**	**p**	**aOR (95% CI)**	**p**	**aHR (95% CI)**	**p**
Group								
Duloxetine monotherapy	Reference		Reference		Reference		Reference	
Duloxetine + Amitriptyline	0.64 (0.16, 2.49)	0.522	0.99 (0.53, 1.87)	0.987	0.78 (0.09, 6.35)	0.813	0.67 (0.34, 1.33)	0.255
Duloxetine + Gabapentin	0.81 (0.19, 3.26)	0.763	0.94 (0.49, 1.80)	0.848	0.56 (0.07, 4.09)	0.562	0.96 (0.48, 1.91)	0.909
Serum Creatinine	5.11 (0.16, 181.21)	0.358	0.62 (0.13, 3.09)	0.561	0.04 (0.00, 4.95)	0.193	5.37 (1.02, 28.17)	***0.047***
Hb at baseline	0.86 (0.56, 1.30)	0.476	0.97 (0.80, 1.18)	0.772	0.65 (0.40, 1.01)	0.060	0.96 (0.83, 1.11)	0.580
Family history	0.27 (0.04, 1.28)	0.135	1.71 (0.91, 3.20)	0.095	13.86 (1.02, 254.76)	0.050	0.96 (0.40, 2.34)	0.935
Chemotherapy duration >6mo	0.36 (0.09, 1.37)	0.135	1.07 (0.60, 1.90)	0.824	0.02 (0.00, 0.22)	***0.003***	0.80 (0.30, 2.11)	0.654

Logistic regression models estimated the association between treatment group and the likelihood of achieving ≥30% reduction in pain score, expressed as adjusted odds ratios (aOR) with 95% confidence intervals (CI). Cox proportional hazards models evaluated the time to achieving ≥30% pain reduction and are presented as adjusted hazard ratios (aHR) with 95% CI. Covariates included in the subgroup models were selected from variables showing statistically significant differences between strata in Supplementary Table S4, while variables with rare occurrences or extremely unbalanced distributions were excluded to maintain model stability after stratification. Reference categories: Duloxetine monotherapy for treatment group. BMI: Body mass index; Hb: Hemoglobin.

Table S6. Interaction analyses evaluating effect modification by sex and continuous age on the association between treatment group and pain reduction outcomes

**Interaction of treatment group with sex models**				
**Variable**	**Logistic regression aOR (95% CI)**	**p**	**Cox regression aHR (95% CI)**	**p**
Group				
Duloxetine monotherapy	Reference		Reference	
Duloxetine + Amitriptyline	0.54 (0.15, 1.83)	0.328	1.16 (0.69, 1.94)	0.578
Duloxetine + Gabapentin	0.72 (0.21, 2.37)	0.591	1.08 (0.64, 1.82)	0.786
Sex (Male vs Female)	0.52 (0.07, 2.49)	0.451	1.44 (0.74, 2.78)	0.278
Interaction				
Duloxetine + Amitriptyline × Male sex	2.93 (0.36, 31.24)	0.333	0.48 (0.20, 1.15)	0.099
Duloxetine + Gabapentin × Male sex	4.44 (0.60, 44.39)	0.164	0.55 (0.23, 1.32)	0.180
**Interaction of treatment group with continuous age models**				
**Variable**	**Logistic regression aOR (95% CI)**	**p**	**Cox regression aHR (95% CI)**	**p**
Group				
Duloxetine monotherapy	Reference		Reference	
Duloxetine + Amitriptyline	0.84 (0.02, 27.67)	0.923	1.01 (0.21, 4.95)	0.986
Duloxetine + Gabapentin	0.73 (0.02, 22.58)	0.859	0.60 (0.12, 3.17)	0.551
Age (Per 1-unit increase in Age)	0.98 (0.93, 1.03)	0.449	1.01 (0.99, 1.04)	0.279
Interaction				
Duloxetine + Amitriptyline × Age	1.00 (0.93, 1.08)	0.975	1.00 (0.97, 1.03)	0.848
Duloxetine + Gabapentin × Age	1.01 (0.94, 1.09)	0.742	1.01 (0.98, 1.04)	0.650

Interaction terms were introduced into both logistic regression and Cox proportional hazards models to assess whether the treatment effect differed according to sex or continuous age. Results are presented as adjusted odds ratios (aOR) for logistic regression and adjusted hazard ratios (aHR) for Cox regression with 95% confidence intervals (CI). Age was modeled as a continuous variable in the interaction models. The reference category for treatment group was duloxetine monotherapy, and the reference category for sex was female. Non-significant interaction terms indicate no evidence of effect modification of the treatment effect by sex or age.

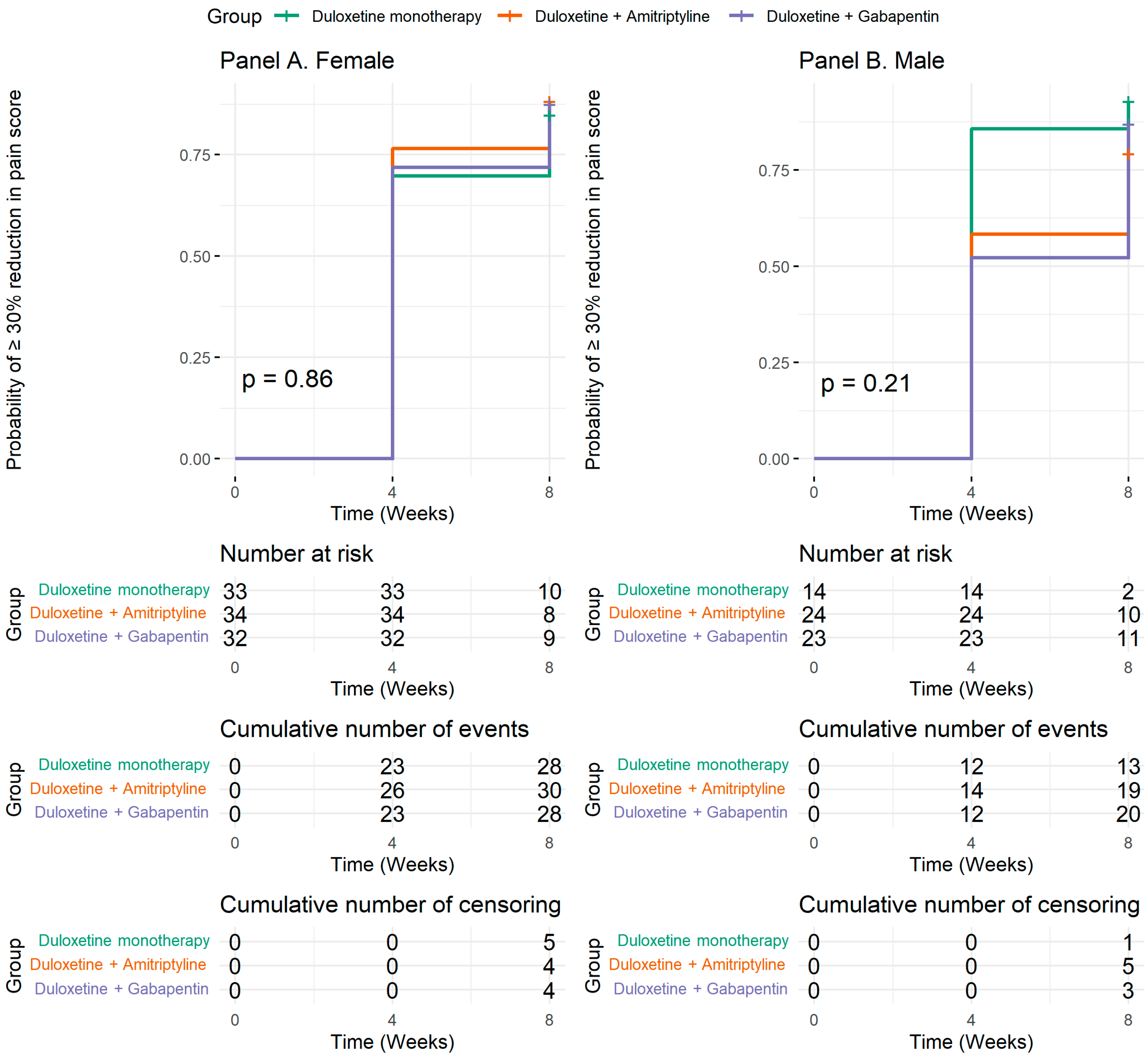

Figure S1. Kaplan–Meier curves of time to ≥30% pain reduction stratified by sex. Kaplan–Meier curves illustrating the probability of achieving ≥30% reduction in pain score during the 8-week follow-up according to treatment group within each sex subgroup. Panel A shows results for female patients, and Panel B shows results for male patients. Curves correspond to duloxetine monotherapy, duloxetine plus amitriptyline, and duloxetine plus gabapentin. The numbers at risk, cumulative events, and censoring counts are displayed beneath each panel. Log-rank tests demonstrated no statistically significant differences between treatment groups within either sex subgroup

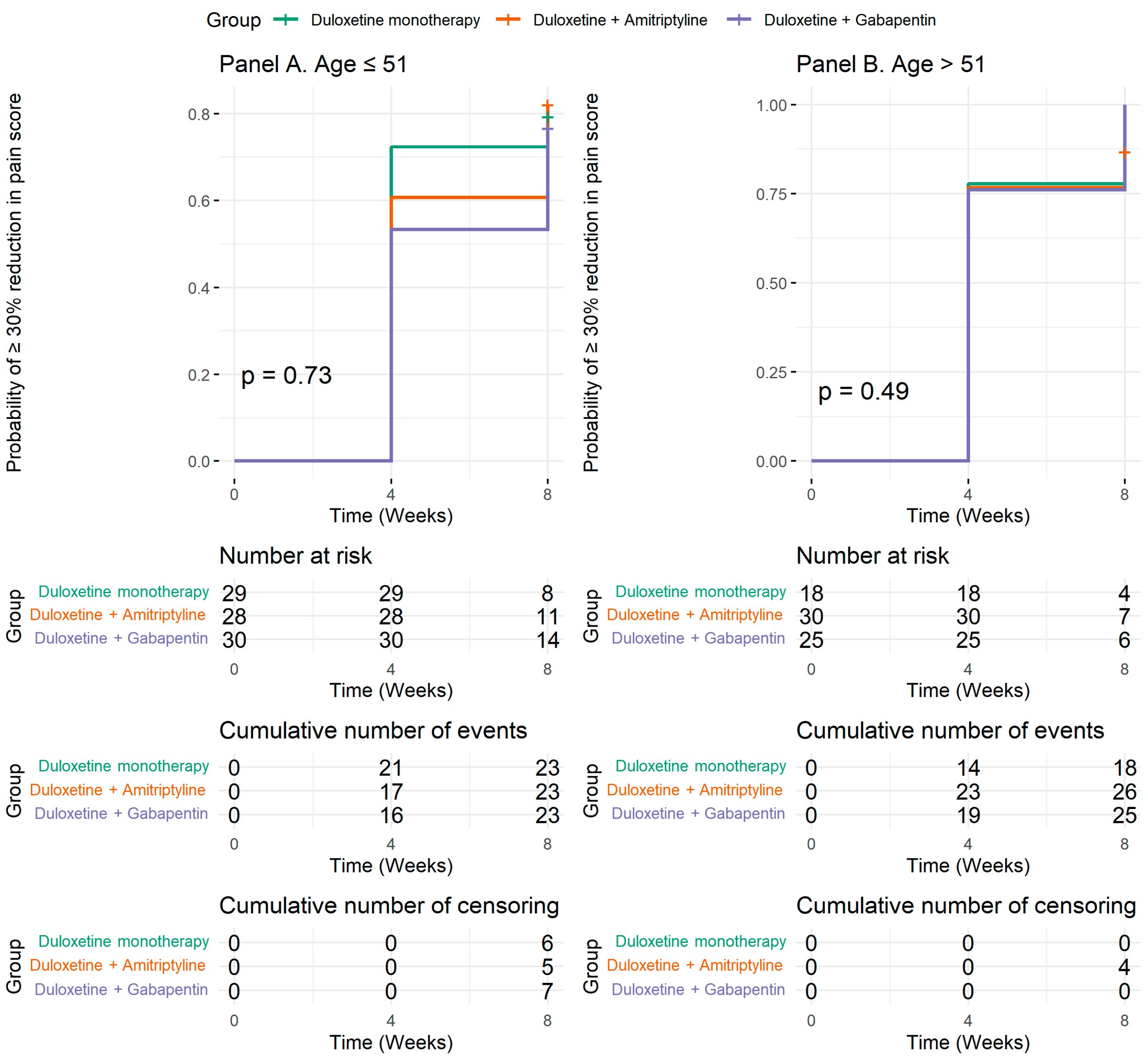

Supplementary Figure S2. Kaplan–Meier curves of time to ≥30% pain reduction stratified by age group. Kaplan–Meier curves showing the probability of achieving ≥30% reduction in pain score over the 8-week follow-up period according to treatment group within each age subgroup. Panel A represents patients aged ≤51 years, and Panel B represents patients aged >51 years. Curves are shown for duloxetine monotherapy, duloxetine plus amitriptyline, and duloxetine plus gabapentin. The numbers at risk, cumulative events, and censoring counts are displayed below each panel. Log-rank tests indicated no statistically significant differences between treatment groups within either age subgroup.

Table S7. Concomitant and rescue analgesics used during the study

**Analgesic category**	**Agent documented in study**	**Baseline use in main manuscript, n (%)**	**Duloxetine monotherapy n (%)**	**Duloxetine + Amitriptyline n (%)**	**Duloxetine + Gabapentin n (%)**	**Dose/frequency**
Opioid	Tramadol	69/160 (43.1)	20/47 (42.6)	22/58 (37.9)	27/55 (49.1)	50-100mg

Analgesic category	Agent documented in study	Baseline use in main manuscript, n (%)	Duloxetine monotherapy n (%)	Duloxetine + Amitriptyline n (%)	Duloxetine + Gabapentin n (%)	Dose/frequency
analgesic						Regular or PRN
Non-opioid analgesic	Paracetamol (acetaminophen)	43/160 (26.9)	12/47 (25.5)	12/58 (20.7)	19/55 (34.5)	1000 mg Prn or regular
